# Investigation of antibody dependent cellular cytotoxicity as a mechanism of action for a novel anti-PD-L1 monoclonal antibody

**DOI:** 10.1186/2051-1426-2-S3-P96

**Published:** 2014-11-06

**Authors:** Benjamin Boyerinas, Caroline Jochems, Kenneth W Hance, Christopher R Heery, James L Gulley, Helen Sabzevari, Jeffrey Schlom, Kwong Tsang

**Affiliations:** 1 Laboratory of Tumor Immunology and Biology, CCR, NCI, NIH, Bethesda, MD, USA; 2 EMD Serono Research & Development Institute, Inc., Billerica, MA, USA; 3 CCR, NCI, NIH, Bethesda, MD, USA

## Purpose

Expression of the immune checkpoint protein PD-L1 constitutes a major mechanism of tumor immune evasion. Multiple clinical trials in solid tumors have demonstrated that inhibition of tumor PD-L1 or immune effector PD-1 via monoclonal antibodies (mAbs) can produce dramatic clinical responses in many cancer patients. The main function of these mAbs is to inhibit signaling induced by ligation of PD-L1 on tumor cells with PD-1 on tumor infiltrating immune effectors. Antibody-dependent cellular cytotoxicity (ADCC) represents an additional mechanism of action for mAbs of the I_g_G1 isotype. In the current study, we describe investigations of a novel anti-PD-L1 mAb of the I_g_G1 isotype (MSB0010718). This mAb is currently in Phase I clinical trials for patients with metastatic or locally advanced solid tumors at the NCI, and is the first such mAb with the capacity to induce ADCC of PD-L1 positive tumor cells. We sought to investigate MSB0010718's ability to induce ADCC and to determine factors affecting tumor cell sensitivity to this mechanism.

## Results

Using whole PBMCs as effectors in *in vitro *ADCC assays, we demonstrated that many cancer cell lines are sensitive to ADCC induced by MSB0010718. Sensitivity to ADCC positively correlated with PD-L1 MFI as determined by flow cytometry. Treatment of tumor cell lines with IFN-γ increased PD-L1 expression with concurrent increase in ADCC sensitivity in some cases. Isolation of NK cells for use as effectors significantly increased ADCC activity as compared to whole PBMCs, demonstrating NK cells as the major effectors of ADCC. ADCC activity could be significantly increased via activation of NK effectors with IL-12, suggesting potential synergy with IL-12 based therapeutics. Furthermore, a MUC1^+ ^tumor cell line that was insensitive to CD8^+ ^MUC1-specific CTL was demonstrated to be sensitive to the ADCC mechanism as a result of high surface PD-L1 expression.

## Conclusions

As it is clear that not all patients respond to current PD-1 or PD-L1 based therapies, additional mechanisms of action may be needed to increase therapeutic efficacy. Our data demonstrate significant ADCC activity induced via MSB0010718. This mechanism of action represents a potential advantage for MSB0010718 over other anti-PD-L1 mAbs, as it can induce target cell lysis in the absence of an effective CD8^+ ^CTL response. As cell surface PD-L1 density was an important predictor of ADCC sensitivity, this mechanism is expected to be most active against tumors with high density PD-L1 expression.

